# Editorial: Insights in nutritional epidemiology

**DOI:** 10.3389/fnut.2022.1032825

**Published:** 2022-09-27

**Authors:** Mauro Serafini, Francesco Sofi, Megan A. McCrory

**Affiliations:** ^1^Faculty of Biosciences and Technologies for Agriculture, Food and Environment, University of Teramo, Teramo, Italy; ^2^Department of Experimental and Clinical Medicine, University of Florence, Florence, Italy; ^3^Department of Health Sciences, Boston University, Boston, MA, United States

**Keywords:** epidemiology, nutrition, obesity, health, degenerative diseases, dietary assessment, biomarkers

We are now entering the third decade of the twenty-first century, and, especially in the last years, the achievements made by scientists have been exceptional, leading to major advancements in the fast-growing field of Nutritional Epidemiology. Frontiers has organized a series of Research Topics to highlight the latest advancements in nutrition in order to be at the forefront of science in different fields of research. This editorial initiative of particular relevance was focused on new insights, novel developments, current challenges, latest discoveries, recent advances and future perspectives in the field of Nutritional Epidemiology. Specifically, the Research Topic solicited brief, forward-looking contributions describing the state of the art, outlining, recent developments and major accomplishments that have been achieved and that need to occur to move the field forward. The goal of this special edition Research Topic was to shed light on the key advancement achieved in the past decade in the field of nutritional epidemiology with a special focus on the role of diet in improving human health, and on its future challenges to provide a thorough overview of the field and to identify novel research avenues.

Regarding cancer, Zhong et al. aimed to understand whether fried food consumption is associated with the risk of pancreatic cancer in a population-based cohort of 101,729 US adults. The review of Fan Y. et al. provides an overview of the current burden, underlying risk factors, and co-existence of malnutrition and other infections including cancer.

In the area of aging, the article by Angelino et al. highlights, for the first time, the importance of meal timing and of a daily caloric restriction lapse, in human longevity.

The paper by Li et al. (A) and corrigendum Li et al. (B) analyzed the trends of obesity and metabolic status among Chinese population in 2012–2020. The work by He et al. focuses on the relationship between serum serine levels and all-cause mortality in general hypertensive patients in a longitudinal cohort. Liu M. et al. evaluate the association between dietary selenium intake and the risk of kidney stones in 30,184 adults.

Fan L. et al. evaluated China's achievements in iodine deficiency disorders (IDDs) prevention. Yang et al. performed a two-sample Mendelian randomization (MR) analysis to evaluate the association between serum vitamin D levels and atrial fibrillation (AF) risks. The work by Liu H. et al. was aimed to determine the prevalence of malnutrition in tertiary hospitals of China and the associations between malnutrition and adverse clinical outcomes. Zahidi et al. found that food insecurity was associated with common mental health problems (CMHPs) among a sample of reproductive-aged women in Kabul, Afghanistan. Liu Y. et al. explore the relationship between serum 25(OH)D, cadmium, and CRP with all-cause mortality among people in diabetic and non-diabetic individuals in the NHANES. Also the work by Lin et al. utilized data from NHANES study to evaluate the relationship between natural yogurt and dietary probiotic supplements consumption and mortality in US adults. In the epigenetic field, Zhou et al. examine whether the associations between plasma selenium and 3-year lipid changes is modified by rs7579 polymorphism. Song et al. aimed to evaluate the effect of vitamin D on the risk of vitiligo using meta-analysis and Mendelian randomization (MR). Lastly, Orsini et al. investigated the interaction analysis based on Shapley values and extreme gradient boosting in a large epidemiological prospective study.

In conclusion, we hope that this article collection will inspire, inform and provide direction and guidance to researchers in the field of nutritional epidemiology.

## Author contributions

MS wrote the initial draft of the manuscript. FS and MM finalized the manuscript. All authors contributed to the article and approved the submitted version.

## Conflict of interest

The authors declare that the research was conducted in the absence of any commercial or financial relationships that could be construed as a potential conflict of interest.

## Publisher's note

All claims expressed in this article are solely those of the authors and do not necessarily represent those of their affiliated organizations, or those of the publisher, the editors and the reviewers. Any product that may be evaluated in this article, or claim that may be made by its manufacturer, is not guaranteed or endorsed by the publisher.

